# The Resilience of South African School Teachers in the Time of COVID-19: Coping with Risk of Infection, Loneliness, and Anxiety

**DOI:** 10.3390/ijerph20043462

**Published:** 2023-02-16

**Authors:** Anita Padmanabhanunni, Tyrone Pretorius

**Affiliations:** Department of Psychology, University of the Western Cape, Bellville 7530, South Africa

**Keywords:** perceived infectability, germ aversion, resilience, loneliness, anxiety, path analysis

## Abstract

The COVID-19 pandemic precipitated an overall increase in the global prevalence of mental health disorders and psychological distress. However, against this backdrop, there was also evidence of adaptation and coping, which suggested the influence of protective factors. The current study aims to extend previous research on the role of protective factors by investigating the health-sustaining and mediating roles of resilience in the relationship between perceived vulnerability to disease, loneliness, and anxiety. Participants consisted of a convenience sample of schoolteachers (N = 355) who completed the Perceived Vulnerability to Disease Questionnaire, the short form of the Connor–Davidson Resilience Scale, the University of California, Los Angeles Loneliness Scale, and the trait scale of the Spielberger State-Trait Anxiety Scale, through an online link created with Google Forms. The results of path analysis indicated significant negative associations between resilience and both loneliness and anxiety. These results indicate the health-sustaining role of resilience. In addition, resilience mediated the relationships between germ aversion and perceived infectability, on the one hand, and loneliness and anxiety, on the other hand. The findings confirm that resilience can play a substantial role in counteracting the negative impact of the pandemic on mental health.

## 1. Introduction

Public health responses to the COVID-19 pandemic, including national lockdowns, restrictions on travel, closure of educational institutions, and stay-at-home orders, have disrupted the lives of people on a global scale [1]. Recent studies have confirmed that these pandemic-related stressors and the associated life changes lead to serious psychological distress and common mental health disorders, including increased loneliness and anxiety [2,3]. Loneliness is defined as a distressing emotional state arising from an unmet need for social connection [4]. It is characterized by negative thoughts of being alone, isolated, and disconnected from others [5]. In a systematic review and meta-analysis of longitudinal studies on loneliness, Ernst and colleagues [6] concluded that overall levels of loneliness increased during the pandemic. Similar results have been reported in other systematic reviews and meta-analytic studies focusing on distinct population groups (e.g., older adults [7]; children and adolescents [8]).

It has been suggested that extended periods of social isolation and physical social distancing due to pandemic containment measures underlie the elevated levels of loneliness experienced by people in different countries (UK [9]; Middle East [10]; USA [11]; Japan [12]). Even before the pandemic, loneliness was identified as a significant public health concern due to its association with adverse physical and psychological health outcomes [13]. Persistent loneliness has been shown to heighten the risk for hypertension and coronary heart disease, leading to increased mortality. Loneliness has also been related to reduced capacity for emotional self-regulation and greater incidence of mental health problems—particularly depression, anxiety, and suicide risk [13,14]. Perceived feelings of loneliness have been identified as a specific risk factor for anxiety and chronic stress [15]. Studies have suggested that individuals experiencing feelings of loneliness typically have fewer meaningful social connections, which enhances their risk of anxiety when they encounter stressors [16]. In the context of COVID-19, heightened anxiety levels have been associated with concerns about the wellbeing of family members, worries about the risk of infection, and uncertainty about the course of the pandemic and economic downturn [17].

Due to the rapid and unprecedented spread of COVID-19 as well as high mortality rates, the current pandemic has generated a widespread sense of vulnerability to infection. A multi-site study that included national samples in 10 countries across Europe, America, and Asia concluded that perceived susceptibility to infection and worries about the likelihood of family members or oneself contracting the virus were significantly high during the initial stages of the pandemic [18]. Perceived vulnerability is defined as the individual’s subjective cognitive appraisal of the likelihood of being infected and is characterized by both cognitive (risk perception) and emotional (e.g., fear, worry, aversion, or disgust) elements [19].

Although greater perceptions of vulnerability to infection may serve an adaptive function by encouraging the adoption of self-protective behaviors (e.g., mask wearing and social distancing), heightened and persistent appraisals of vulnerability can undermine people’s sense of safety and control and thereby aggravate anxiety. Existing studies have reported associations between perceived vulnerability to disease, germ aversion and general anxiety [20]. While perceived vulnerability to disease refers to the subjective susceptibility to disease, germ aversion entails feelings of discomfort in situations where germs may be transmitted [21,22]. It has been suggested that individuals with higher levels of susceptibility to disease and germ aversion are more likely to overestimate the severity and likelihood of infection and thereby experience heightened levels of anxiety [22,23]. This, in turn, is likely to lead to increased avoidance, social withdrawal, and isolation, thereby aggravating loneliness and undermining mental wellbeing [18].

In contrast to studies that have demonstrated substantial increases in mental health problems and psychological distress during the pandemic, there has also been increasing evidence that many people were able to cope effectively with the outbreak and its containment measures [24,25]. For example, Fancourt and colleagues [26] reported high levels of anxiety and depression among adults in England during the initial outbreak and lockdown restrictions but observed that these rates declined rapidly. In a U.S. study, Daly and Robinson [27] found increased rates of psychological distress during the first wave of the pandemic, before subsequently declining to baseline levels. González-Sanguino and colleagues [28] reported that following the easing of confinement measures in Spain, depressive symptoms rapidly declined after a substantial initial increase. These studies have provided evidence suggesting that there is increased resilience in the general population. Psychological resilience is characterized by positive adaptation to adverse events, the ability to recover from challenging stressors, and the capacity to adapt to change [29].

Existing studies have identified a cluster of factors that confer resilience, including social support, active problem-solving skills, self-efficacy, a sense of self-control, and self-compassion [30]. Research on resilience during the pandemic has focused on its mediating role in, for example, the associations between stress and burnout [31], stress and quality of life [32], social isolation and wellbeing [33], COVID-19-related anxiety and fear of COVID-19 [34], and psychological distress and loneliness [35]. Resilience and fear of being infected have been reported to be among the most salient predictors of mental health status [36]. Furthermore, psychological resilience has been found to be critical to countering the detrimental effects of heightened appraisals of susceptibility to infection [36].

The current study thus aims to extend research on the role of resilience in the context of the COVID-19 pandemic by examining its health-sustaining and mediating roles in the relationship between perceived vulnerability to disease and both loneliness and anxiety among a sample of South African school teachers.

In South Africa and elsewhere, COVID-19-related containment measures included the closure of all educational institutions and the resultant transition to online learning. In South Africa, this occurred from March to August 2020. During that time, teachers had to manage a significantly increased workload, changes to their daily routines, and challenges associated with working from home (e.g., child are and homeschooling for their own children) [37]. Teachers had to negotiate the rapid transition to remote online teaching and the implementation of digital pedagogies. Many teachers had to master new information community technology (ICT) relatively quickly and guide students and parents in the use of online modalities. The effectiveness of online learning is grounded in sufficient planning and the development and testing of online instructional programs. In the context of the pandemic, the unexpected and unprecedent migration to online educational instruction contributed to stress and anxiety among both teachers and students. A significant disadvantage of online teaching is the lack of direct interaction and engagement between teachers and students. Instructional platforms such as live Zoom lectures have been appraised by students are accessible but not necessarily effective in promoting learning and as less enjoyable compared to direct interactions [38]. Technology-mediated learning and teaching is not feasible in contexts where there is limited access to digital technologies. In such instances, students may not be able to engage academically and this can contribute to anxiety and despondency [38].

Studies have confirmed that the disruption to the educational system significantly impacted teachers’ mental health and wellbeing on a global scale and contributed to burnout, heightened levels of anxiety and depression, and teacher attrition [39]. However, there has also been evidence that some teachers were able to effectively negotiate these complex stressors. Walter and Fox [40], for example, reported that teachers who were able to maintain a sense of connection with their school community and worked within supportive administrative and leadership teams had a greater sense of self-efficacy and were better able to cope with the aforementioned challenges.

By August 2020, the South African government mandated a return to conventional schooling due to disparities in access to education [41]. Many schools across South Africa are located in disadvantaged communities or rural settings where the resources to engage in digital learning are not available. The return to traditional schooling was found to increase teachers’ fears and anxieties about exposure to COVID-19 and possibly transmitting it to their loved ones [41]. Appraisals of enhanced susceptibility to infection were likely to further aggravate these fears and enhance psychological distress. As teachers entered the 2021 and 2022 school years, they were tasked with the challenge of addressing the substantial learning loss that had arisen due to school closures, the need to maintain high standards of teaching, and the implementation of COVID-19-related safety protocols.

Given the distinctive impact of the pandemic and its containment measures on schoolteachers, it remains imperative to investigate psychological factors that mediate negative mental health outcomes and promote wellbeing. This study is grounded in the protection motivation theory (PMT) [42], a social cognitive theory that aims to understand how individuals respond to threats to their health and safety. According to PMT, cognitive appraisals of both threats and safety shape an individual’s motivation and intention to engage in protective behaviors. Appraisals of threats are derived from an individual’s beliefs about the risk to their health status and are based on environmental (e.g., exposure to the media) and personal (e.g., prior experiences) factors. When an individual perceives a threat (e.g., threat of exposure to COVID-19) to be serious, they engage in a coping response appraisal process by assessing the resources available to cope with or prevent the threat and their capacity to implement a coping response. This latter type of appraisal is similar to the construct of self-efficacy (i.e., individuals’ belief in their abilities) and has the potential to confer psychological resilience and mitigate the development of adverse mental health outcomes, including loneliness and anxiety.

To date, most research on resilience in the context of the COVID-19 pandemic has investigated its direct effect on mental health outcomes (e.g., [43,44]). Comparatively fewer studies have examined resilience’s protective role in the relationship between heightened perception of vulnerability to disease and psychological distress. The current study aims to address this gap in the literature.

Based on the evidence of a direct relationship between resilience and psychological distress (as summarized above), we hypothesized, with respect to the health-sustaining role of resilience, that resilience is negatively associated with anxiety and loneliness. Furthermore, with respect to the mediating role of resilience, we hypothesized that resilience mediates the following relationships: perceived infectability and loneliness, perceived infectability and anxiety, germ aversion and loneliness, and germ aversion and anxiety.

## 2. Materials and Methods

### 2.1. Participants

Participants consisted of a convenience sample of schoolteachers (N = 355). Data collection occurred from April to July 2021. During this timeframe, the Delta variant of COVID-19 was the dominant strain in South Africa [45], and the South African government consequently increased lockdown regulations to the second strictest level of lockdown [46]. It was therefore not possible to conduct random sampling; in addition, South Africa’s legislation regarding the protection of personal information prevented us from accessing national databases of teachers and thereby obtaining a sampling frame. The majority of the participants in the sample resided in an urban area (61.7%), were women (76.6%), and taught at the primary school level (grades 1–7; 61.1%). The mean age of the sample was 41.89 years (SD = 12.4; range = 23 to 73) and the mean number of years in the teaching profession was 15.7 years (SD = 11.7; range = 1 to 48). The demographics of the sample aligned very closely with population data reported in an international teaching and learning survey that reported that 60% of teachers in South Africa are women, with a mean age of 43, and a mean working experience of 15 years [47].

### 2.2. Instruments

Participants completed the following instruments: the Perceived Vulnerability to Disease Questionnaire (PVD-Q: [21]), the short form of the Connor-Davidson Resilience Scale (CD-RISC-10: [48]), the University of California, Los Angeles Loneliness Scale (UCLA-LS: [4]), and the trait scale of the Spielberger State-Trait Scale (STAI-T: [49]). In addition, participants completed a brief demographic questionnaire.

The PVD-Q consists of 15 items on a 7-point scale, ranging from “strongly disagree” to “strongly agree”. It measures two separate dimensions associated with perceptions of vulnerability to disease: perceived infectability (PI), which refers to the subjective susceptibility to disease, and germ aversion (GA), which refers to feelings of discomfort in situations where germs may be transmitted. Duncan and colleagues [21] provided evidence for the convergent, discriminant, and predictive validity of the two substrates and reported satisfactory indices of internal consistency (PI: α = 0.87; GA: α = 0.74). Recent studies have similarly reported satisfactory indices of internal consistency (e.g., PI = 0.78, GA = 0.72 [50]; PI = 0.92, GA = 0.74 [51]), with the GA subscale consistently demonstrating lower reliability than the PI subscale.

The CD-RISC-10 is a short form of the original 25-item resilience scale developed by Connor and Davidson [52]. As the name indicates, it consists of 10 items scored on a 5-point scale that ranges from “not true at all” (0) to “true nearly all of the time” (4). In the original validation of the 10-item version, Campbell-Sills and Stein [48] provided evidence for the unidimensionality and construct validity of the scale and reported a reliability coefficient of 0.85. The scale has been used in a wide variety of contexts with similar sound estimates of internal consistency; for example, Cheng and colleagues [53] used the CD-RISC-10 in China with undergraduate students and depressive patients and reported reliability coefficients between 0.88 and 0.92. Blanco and colleagues [54] used the scale in Spain with non-professional caregivers and reported a reliability coefficient of 0.85. In South Africa, Pretorius and Padmanabhanunni [55] used item-response theory and classical test theory to confirm the dimensionality and validity of the CD-RISC-10 and reported very satisfactory estimates of reliability (α = 0.95, composite reliability = 0.96, Mokken scale reliability = 0.95).

The UCLA-LS is a 20-item measure of the subjective experience of loneliness and is scored on a 4-point scale ranging from “never” to “often”. Russell and colleagues [4] provided evidence for the convergent and construct validity of the scale and reported reliability coefficients ranging between 0.89 and 0.94. The UCLA-LS is the most widely used measure of loneliness and has been used in several different countries. For example, in Taiwan, Chung-Ying and colleagues [56] used the scale with sexual-minority men and reported a Cronbach’s alpha of 0.92 and a McDonald’s omega of 0.94. A systematic review of the UCLA-LS found that reliability coefficients for the scale ranged between 0.71 to 0.95 [57]. Pretorius [58,59] reported reliability coefficients of 0.81 and 0.92 in two different studies in South Africa.

The STAI-T is a 20-item measure of trait anxiety. Responses to the items are made on a 4-point scale ranging from “almost never” to “almost always”. The author of the scale reported reliability coefficients of 0.84 for men and 0.76 for women [49]. In a reliability generalization study, Barnes and colleagues [60] reported mean reliability coefficients of 0.89 with reliability coefficients ranging between 0.72 and 0.96 across 46 studies. Padmanabhanunni and Pretorius reported a reliability coefficient of 0.88 for the STAI-T when used with South African students [61].

### 2.3. Procedure

Google Forms was used to create an electronic version of the survey. We requested permission from the administrators of Facebook groups of teachers to post the link to the survey on their sites.

### 2.4. Ethics

The study was conducted according to the guidelines of the Declaration of Helsinki and was approved by the research ethics committee of the University of the Western Cape (ethics reference number HS21/3/8; 14 May 2021). Participants provided informed consent on the landing page of the link; they were assured of their anonymity and reminded that participation was voluntary.

### 2.5. Data Analysis

**Figure 1:**
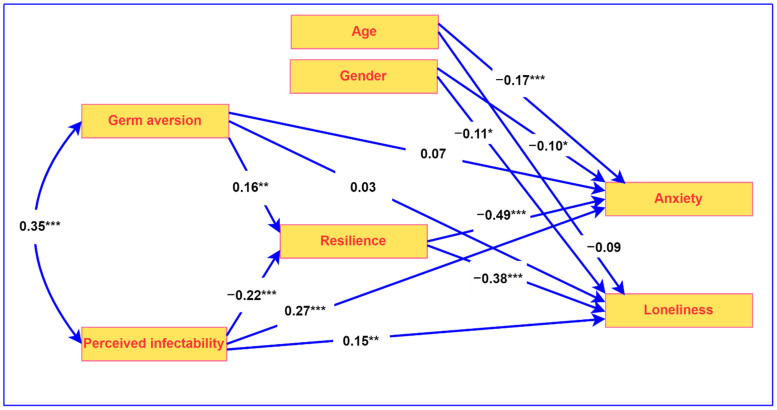
Path analysis model of the mediating role of resilience (*** *p* < 0.001, ** *p* < 0.01, * *p* < 0.05).

Descriptive statistics (means and standard deviations), intercorrelations between variables (Pearson’s correlation), and reliabilities (alpha and omega) were obtained using IBM SPSS for Windows version 28 (IBM Corp., Armonk, NY, USA). Omega was determined using the OMEGA macro developed by Hayes and Coutts for SPSS [62]. Path analysis was conducted with IBM Amos for Windows version 27. Maximum likelihood estimation with bootstrapped 95% confidence intervals was used (see Figure 1). In the path analysis, gender and age were added as covariates.

## 3. Results

The intercorrelations between variables, the reliability coefficients, and the descriptive statistics are reported in Table 1. All the scales demonstrated satisfactory reliability (α and ω = 0.78–0.95), with the exception of the Germ Aversion scale, which demonstrated moderate reliability (α = 0.65, ω = 0.66). Given the number of correlations, we used the Benjamini–Hochberg Procedure to decrease the false discovery rate and thus ensuring we identify significant correlations that could have occurred by chance. The corrected *p*-values are reported above the diagonal and the correlation coefficients below the diagonal.

The *p*-values above the diagonal in Table 1, shows that significant correlations remained significant after the Benjamini-Hochberg correction. Perceived infectability was positively related to both loneliness (r = 0.22, *p* < 0.001) and anxiety (r = 0.38, *p* < 0.001). Thus, high levels of perceived infectability were associated with high levels of loneliness and anxiety. Germ aversion was only positively related to anxiety (r = 0.13, *p* < 0.001), which indicates that high levels of germ aversion were associated with high levels of anxiety. Resilience was negatively related to loneliness (r = −0.40, *p* < 0.001) and anxiety (r = −0.53, *p* < 0.001), indicating that high levels of resilience are associated with low levels of loneliness and anxiety.

The path analysis model that was used to examine the mediating role of resilience is presented in Figure 1, along with the associated standardized regression coefficients. In this model, perceived infectability and germ aversion are the predictors that are antecedents to resilience, while loneliness and anxiety are the dependent variables. Gender and age were significantly related to the predictor variables. In particular, women reported higher levels of anxiety (*M* = 46.03, *SD* = 10.46, *t* = 3.76, *p* < 0.001) and loneliness (*M* = 48.30, *SD* = 11.60, *t* = 3.49, *p* < 0.001) than men (anxiety: *M* = 41.24, *SD* = 8.89; loneliness: *M* = 43.40, *SD* = 9.95). Younger participants reported higher levels of anxiety (*r* = −0.19, *p* < 0.001) and loneliness (*r* = −0.12, *p* = 0.030) than older participants. Gender and age were therefore added to the path analysis model as covariates.

The direct and indirect effects related to the role of resilience are reported in Table 2. The results in Table 2 provide support for all the hypotheses that relate to the role of resilience.

The results reported in Table 2 indicate that resilience was negatively associated with loneliness (β = −0.35, *p* = 0.001, 95% CI [−0.44, −0.29]), and anxiety (β = −0.46, *p* = 0.001, 95% CI [−0.56, −0.42]). The above two hypotheses confirm the health-sustaining role of resilience. In addition, the results in Table 2 show that resilience mediated the relationships between perceived infectability and loneliness (β = 0.08, *p* = 0.001, 95% CI [0.06, 0.15]), perceived infectability and anxiety (β = 0.10, *p* = 0.001, 95% CI [0.06, 0.15]), germ aversion and loneliness (β = −0.06, *p* = 0.001, 95% CI [−0.12, −0.03]), and germ aversion and anxiety (β = −0.07, *p* = 0.002, 95% CI [−0.15, −0.04]).

The results in Table 2 also reflect that resilience was related to both perceived infectability (β = −0.22, *p* = 0.001, 95% CI [−0.31, −0.13]) and germ aversion (β = 0.16, *p* = 0.001, 95% CI [0.07, 0.24]). However, in the case of germ aversion, this was a positive association, indicating that high levels of germ aversion were related to high levels of resilience.

## 4. Discussion

The COVID-19 pandemic precipitated an overall increase in the global prevalence of mental health disorders and psychological distress. Pronounced increases in depressive symptoms, fear and anxiety, loneliness, and stress were documented in various countries and across diverse population groups (e.g., [2,3,36]). However, against this backdrop, there was also evidence of adaptation and coping, which suggested the influence of protective factors. The current study aimed to extend previous research on the role of protective factors by investigating the health-sustaining and mediating roles of resilience in the relationship between perceived vulnerability and disease, loneliness, and anxiety. There were several important findings.

First, resilience had a health-sustaining role in that it was associated with lower levels of loneliness and anxiety during the COVID-19 pandemic. Existing studies (e.g., [9]) have shown that social isolation due to COVID-19-related containment measures (e.g., home confinement) increased the risk of psychological distress, depression, and anxiety. Longer periods of loneliness are predictive of serious adverse mental health outcomes. However, a sense of being able to adapt to challenging experiences can buffer against these outcomes. Additional factors that have been found to cultivate resilience among school teachers during the pandemic include religiosity, meaningful social connections, good teacher-student relationships, high levels of self-efficacy, vaccination program prioritization, and access to mental health resources [63,64]. The South African government prioritized the vaccination of schoolteachers, potentially reducing appraisals of threats associated with the pandemic and related anxiety. Mansfield and colleagues [65] investigated teaching resilience in South Africa before the pandemic and reported that teachers’ personal resources (e.g., optimism, motivation, and perseverance) and the use of adaptive coping strategies (e.g., active problem solving) were instrumental in enhancing resilience. When applied to the current study, these factors likely contributed to resilience among schoolteachers and reduced psychological distress.

Second, high levels of perceived infectability were associated with high levels of anxiety and loneliness. Perceived infectability was associated with increased concern about the outbreak of disease, heightened levels of fear about infection, and negative mental health outcomes in the form of heightened anxiety and stress [66]. Given the rapid spread and potentially life-threatening nature of COVID-19, it is probable that teachers who appraised their risk of infection as high experienced higher levels of anxiety. Many South African schools are located in under-resourced settings where access to personal protective equipment, running water, and sanitation facilities is limited; this can heighten fears of contracting COVID-19 [22]. Heightened appraisals of vulnerability have been associated with greater sensitivity to environmental cues that suggest danger (e.g., someone coughing) and can trigger interpersonal avoidance behaviors and social withdrawal, thereby limiting meaningful social connection [67]. This, in turn, can produce feelings of loneliness. Most participants in the current study were teachers who taught at the primary school level where class sizes are typically larger. In addition, students are younger at this level and may not fully understand the importance of social-distancing measures and may inconsistently use personal protective equipment. This could potentially heighten teachers’ appraisals of perceived infectability and anxiety.

Third, resilience mediated the relationship between perceived infectability and germ aversion, on one hand, and loneliness and anxiety, on the other hand. This is consistent with existing studies that have found that resilience plays a significant role in counteracting psychological distress related to COVID-19. For example, Lara-Cabrera and colleagues [68] reported that resilience played a mediating role in levels of anxiety, depression, and stress among nurses caring for COVID-19 patients. Kara and Çanakçi [69] similarly found resilience to mediate the relationship between fear of COVID-19 and mental health, while Peker and Cengiz [70] reported that resilience plays a mediating role in the association between happiness and stress in the general population.

A final and distinctive finding of this study was that high levels of germ aversion were associated with high levels of resilience. This stands in contrast with previous studies that have found high levels of perceived vulnerability to disease and germ aversion to be associated with greater stress and adverse psychological outcomes [66,71]. In applying the PMT, it is probable that those with high levels of germ aversion appraise the threat associated with the pandemic as high and thus engage in more protective coping behaviors (e.g., strictly adhering to prevention protocols). For example, germ aversion has been shown to predict social distancing and reduced use of public transportation [72]. These strategies can prevent infection and consequently enhance feelings of safety and control, thereby fostering resilience.

Our findings may have implications for interventions and suggest that enhancing resilience among vulnerable population groups can mitigate adverse mental health outcomes. Currently, there is evidence that inquiry-based stress reduction is an effective intervention in addressing teacher distress and burnout [37]. In low-to-middle income countries, such as South Africa, where public health resources are constrained, more cost-effective strategies such as group therapy and online delivery of short-term interventions may be a more viable option than traditional face-to-face interventions in bolstering resilience. Furthermore, implementing stress management programs in school settings and work–life balance policies may further enhance coping and promote resilience.

This study has certain limitations. First, the findings regarding the causal relationship between variables were limited by the cross-sectional nature of this study’s design. The measures used in this study do not refer to the pandemic specifically and causal interpretations will need to be made with caution. Future studies using longitudinal designs would be needed to confirm the findings on causal directionality. Second, the sample comprised school teachers mostly from one geographical area, and most teachers in the sample were women. This impacted the representativeness and generalizability of the findings. Furthermore, the mean age of teachers in the sample was 42 years and this could have a bearing on risk perception. Older teachers as well as those with co-morbid health conditions are at greater risk of more serious symptoms and this could have a bearing on their risk perception and anxiety levels. Research using more heterogonous samples would be beneficial in replicating and extending the results of this study. Third, the use of an online survey may have favored teachers with access to digital technology and led to response bias. Studies using different methods of data collection may be needed to verify the results of this study. Finally, it is possible that the association between variables could be explained by reverse causality or unobserved factors. Longitudinal research would be beneficial in confirming causal associations.

## 5. Conclusions

The current study provides empirical evidence of the mediating role of resilience in the relationship between perceived vulnerability to disease and psychological outcome variables. The findings confirm that resilience can play a substantial role in counteracting the negative impact of the pandemic on mental health. Resilience is a potentially modifiable characteristic and can be enhanced through intervention efforts that focus on promoting cognitive flexibility, active problem solving, and emotional regulation. Our findings offer further support for the development of resilience-based interventions to protect the mental health and wellbeing of vulnerable populations. Digital interventions such as the Stress Management and Resiliency Training (SMART) program have demonstrated efficacy [73] in improving stress levels, enhancing quality of life, reducing burnout and promoting mindfulness and resilience among schoolteachers. These types of digital interventions are cost effective and relatively easily accessible and may assist teachers in coping with the stressors encountered in their profession.

## Figures and Tables

**Table 1:** Intercorrelations between variables, reliabilities, and descriptive statistics.

Variables and Indices	1	2	3	4	5
1. Germ aversion	—	0.000	0.153	0.368	0.020
2. Perceived infectability	0.35 ***	—	0.003	0.000	0.000
3. Resilience	0.08	−0.17 **	—	0.000	0.000
4. Loneliness	0.05	0.22 ***	−0.40 ***	—	0.000
5. Anxiety	0.13 *	0.38 ***	−0.53 ***	0.65 ***	—
Mean	42.9	28.7	26.9	45.0	47.2
SD	8.4	8.8	8.0	10.3	11.3
Alpha	0.65	0.78	0.95	0.91	0.92
Omega	0.66	0.78	0.95	0.91	0.92

*** *p* < 0.001, ** *p* < 0.01., * p < 0.05

**Table 2:** Results of path analysis of the direct and mediating role of resilience.

Effect	Beta	SE	β	95% CI	*p*
Direct effects					
Perceived infectability → resilience	−0.20	0.05	−0.22	[−0.31, −0.13]	0.001
Germ aversion → resilience	0.15	0.05	0.16	[0.07, 0.24]	0.002
Resilience → loneliness	−0.50	0.07	−0.35	[−0.61, −0.38]	0.001
Resilience → anxiety	−0.59	0.05	−0.46	[−0.69, −0.49]	0.001
Indirect effects					
Perceived infectability → resilience → loneliness	0.10	0.03	0.08	[0.06, 0.15]	0.001
Perceived infectability → resilience → anxiety	0.12	0.03	0.10	[0.07, 0.18]	0.001
Germ aversion → resilience → loneliness	−0.07	0.03	−0.06	[−0.12, −0.03]	0.001
Germ aversion → resilience → anxiety	−0.09	0.03	−0.07	[−0.15, −0.04]	0.002

## Data Availability

The raw data supporting the conclusions of this article will be made available by the authors, without undue reservation.
